# Effectiveness of respiratory care modalities on bio-physiological parameters among COPD patients at a tertiary care hospital in Chennai, India

**DOI:** 10.6026/9732063002001984

**Published:** 2024-12-31

**Authors:** Shabana Nisar Ahamed, Theranirajan Ethiraj, Shankar Shanmugam Rajendran, Anandhi Duraikannu, Sudha Devadoss, Divya Bharathi Jayaraman, Sundari Mani

**Affiliations:** 1 Department of Medical Surgical Nursing, College of Nursing, Madras Medical College, The TN Dr MGR Medical University, Chennai, Tamil Nadu, India; 2 Department of Pediatrics, Madras Medical College, The TN Dr MGR Medical University, Chennai, Tamil Nadu, India; 3 Department of Pediatric Nursing, College of Nursing, Madras Medical College, The TN Dr MGR Medical University, Chennai, Tamil Nadu, India; 4 Department of Medical Surgical Nursing, College of Nursing, Madras Medical College, The TN Dr MGR Medical University, Chennai, Tamil Nadu, India

**Keywords:** Chronic obstructive pulmonary disease (COPD), effectiveness, respiratory care modalities, bio-physiological parameters, quasi-experimental technique

## Abstract

Chronic Obstructive Pulmonary Disease (COPD) is one of the prevalent global health problems, with chronic respiratory symptoms and airflow limitation due to anatomic abnormalities primarily in the airways and alveoli. Therefore it is of interest to evaluate the effectiveness of respiratory care modalities on bio-physiological parameters in COPD patients at a tertiary care hospital in Chennai. Using a quasi-experimental design with pre-test and post-test control groups, 80 subjects were selected through non-probability purposive sampling. The intervention, lasting 10-15 minutes and administered thrice daily over four weeks, aimed to enhance physiological parameters. Results indicated a statistically significant improvement, with the mean respiratory issue score decreasing from 7.45 to 4.80 (p < 0.05). These findings suggest that the respiratory care modalities effectively improved bio-physiological parameters in COPD patients, demonstrating significant benefits from the interventions. Thus, respiratory care can enhance the health status of individuals with COPD.

## Background:

Chronic Obstructive Pulmonary Disease (COPD) is an obliterative and irreversible dysfunction of the lungs with some persistent inflammation [1]. It primarily arises from prolonged exposure to harmful particulates and gases, most commonly from cigarette smoke, although exposure to air pollution, occupational dust and chemicals also play significant roles [2]. The disease typically manifests as emphysema and chronic bronchitis, with symptoms including persistent cough, wheezing, shortness of breath and excessive mucus production [3]. These symptoms often exacerbate over time, leading to increased difficulty in breathing and significantly impacting the quality of life [4]. Therefore, it is of interest to respiratory care modalities such as balloon-blowing therapy, pursed lip breathing exercises and diaphragmatic breathing exercises are pivotal in managing bio physiological parameters in COPD patients [5].

## Methodology:

## Statement of the problem:

The effectiveness of Respiratory Care Modalities on Bio Physiological parameters among COPD patients in tertiary care Centre, Chennai to reported.

## Objectives:

The pre-test levels of bio-physiological parameters in COPD patients from both the interventional and control groups to reported. It will compare the pre-test and post-test levels of these parameters to evaluate the effectiveness of respiratory care modalities in improving health outcomes among the participants. Additionally, the research will explore the association between the post-test levels of bio-physiological parameters and various demographic and clinical variables among COPD patients. Through this comprehensive approach, the study seeks to provide insights into the impact of respiratory interventions on patient health.

## Hypothesis:

H1: There is a notable difference in the post-test levels of bio-physiological parameters among COPD patients when comparing the interventional and control groups.

H2: A significant relationship exists between the post-test levels of bio-physiological parameters in COPD patients from both groups and their selected demographic and clinical characteristics.

## Inclusion criteria:

Participants in this study included patients with Chronic Obstructive Pulmonary Disease (COPD) in stages I and II who were receiving regular treatment. Eligible individuals were between 41 and 70 years of age and had willingly agreed to participate in the research. The inclusion criteria also allowed for patients with or without diabetes mellitus (DM) and hypertension (HTN) provided these conditions were well-managed. Both males and females were included and participants were required to be able to read or write in Tamil or English.

## Exclusion criteria:

Patients with Chronic Obstructive Pulmonary Disease (COPD) were excluded if they had uncontrolled co-morbid conditions. Individuals who regularly performed breathing exercises, such as yoga, or had participated in a pulmonary rehabilitation program within the past six months were also excluded. Additionally, those with a history of psychiatric illness were excluded from the study.

## Ethical consideration:

This was approved by the Institutional Ethics Committee of Madras Medical College on 11.10.2023, where all the protocols underwent ethics in medical research and kept it patient-safe. Moreover, written permission has been obtained from the Director of the Institute of General Medicine, Rajiv Gandhi Government General Hospital, Chennai, as the study itself complies with institutional norms while protecting the rights of the participants.

## Research variables:

## Independent variable:

Respiratory care modalities include Balloon Blowing Therapy, Diaphragmatic breathing and Pursed lips breathing exercises.

## Dependent variable:

Bio-physiological parameters in COPD patients.

## Tools for data collection:

## Section I:

Socio-demographic Questionnaires include Age in years, Sex, Level of Education, Marital Status, Place of employment, Family income, Habits, Family history of respiratory illness, Residence and presence of pet animals in the house.

## Section II:

Clinical Variables include Age in years at onset of COPD and duration of illness, previous hospitalization for Respiratory problems within a year, regular treatment for COPD, co-morbid condition on DM/HTN, regular treatment for comorbidity, Continuous breathing difficulty and seasonal variations of COPD.

## MRC dyspnoea scale:

The MRC dyspnoea scale, developed in 1986 by the Medical Research Council, grades breathlessness into five grades to estimate intensity. Grade 1: Minor breathlessness on strenuous exercise only. Grade 2: Some breathlessness with exertion but does not have to stop for breath or walk more slowly than peers, even when hurrying on level ground or walking upstairs at their own pace. Grade 3: "Signifying a need to stop for breath while walking at their own pace on level ground or stairs." Grade 4: Are severe dyspnoea and the patients stopping to catch their breath after walking 100 meters. Finally, Grade 5 is the most severe level, wherein they will experience dyspnoea when not leaving the house or performing routine activities such as dressing. The grading system classifies grades one as mild, 2 and 3 as moderate and 4 and 5 as severe.

## Scoring interpretation:

Table 1 explains the Scoring interpretation for respiratory assessment: a score of 0 indicates a regular breathing pattern. Scores ranging from 1 to 6 suggest a mild respiratory problem. A score between 7 and 13 reflects a moderate respiratory issue, while scores 14 to 18 indicate a severe respiratory problem. This classification helps evaluate the severity of respiratory difficulties.

## Data collection procedure:

The data collection period was four weeks. The study was conducted after obtaining the Institutional Ethics Committee's approval. The samples were selected using a non-probability purposive sampling technique. Informed consent was taken from all the participants. The purpose of the study was shared with the participants. The pretest questionnaires on socio-demographic and clinical variables, the MRC Dyspnoea Scale and the bio-physiological Parameters Assessment Scale were administered; it took 10-15 minutes. The participants were divided into interventional and control groups. The interventional group was given balloon-blowing therapy, diaphragmatic breathing and pursed lip breathing exercises. The control group received routine care. Post-interventional levels of bio-physiological Parameters were assessed after 21 days.

## Results:

**Figure 1:**
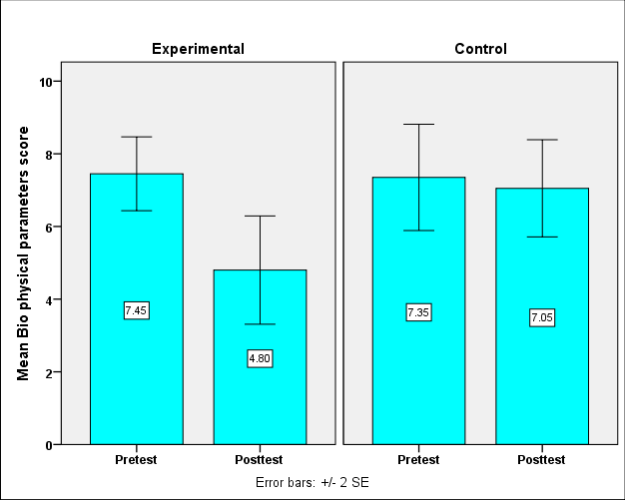
A simple bar with two standard error diagrams compares the women's Dyspnoea scores between interventional and control group

**Figure 2:**
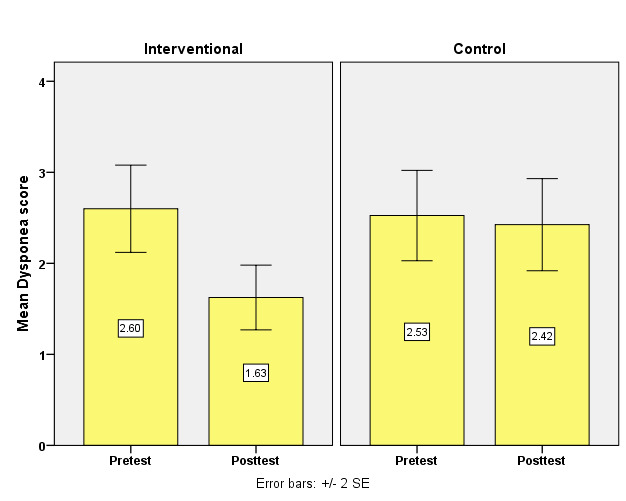
Simple bar with two standard error diagrams compares the bio-physiological parameters score between interventional and control group

Initial bio-physiological parameters showed no significant differences between groups. Post-intervention, significant improvements were noted in the interventional group. There was a statistically significant reduction in the mean respiratory issue score from 7.45 to 4.80 (p < 0.05). Additionally, associations were identified between improved post-test bio-physiological parameters and demographic factors such as age and sex, with younger patients and males showing better outcomes. Table 2 compares pre and post-intervention dyspnoea scores between interventional and control groups. (Figure 1) initially, both groups exhibited similar scores, with the interventional group at a mean of 2.60 (SD = 1.52) and the control group at 2.53 (SD = 1.57), showing no significant difference (p = 0.81). Post-intervention, the interventional group improved significantly, recording a lower mean score of 1.62 (SD = 1.13) compared to the control's 2.42 (SD = 1.60) with a statistically significant difference (p = 0.02). This indicates that the Respiratory Care Modalities effectively reduced dyspnoea severity in the interventional group. Table 3 assesses the bio-physiological parameters scores between the interventional and control groups, both pre and post-intervention, using the Mann-Whitney U-test for analysis. Figure 2 Initially, both groups exhibited similar scores: the interventional group had a pretest mean of 7.45 (SD = 3.21) and the control group had a pretest mean of 7.35 (SD = 4.62) with a negligible mean difference of 0.10 and no significant difference between them (p = 0.83). Post-intervention, the interventional group's mean score improved to 4.80 (SD = 4.71), while the control group's mean was 7.05 (SD = 4.23). The post-test mean difference expanded to 2.25, achieving statistical significance (p = 0.05). This indicates that the Respiratory Care Modalities effectively improved bio-physiological parameters in the interventional group compared to the control group.

## Discussion:

Lalwani *et al.* [6] explored the short-term effects of pursed lip breathing in stable COPD patients. Their findings align with our study, highlighting the positive impact of targeted breathing techniques on respiratory health. Both studies emphasize improved respiratory outcomes, suggesting that structured breathing exercises can significantly enhance patient well-being. Maharem *et al.* [7] compared acupressure and pursed lip breathing techniques, noting both interventions positively affected physiological parameters and dyspnea grades among COPD patients. This study complements our findings by illustrating the effectiveness of various therapeutic approaches, supporting the idea that incorporating different techniques can lead to improved patient outcomes. Mohan *et al.* [8] examined the effects of core stability training on respiratory variables, highlighting the importance of physical exercise in managing respiratory conditions. While their focus was on back pain, the implications for respiratory function reinforce our study's findings that physical interventions can improve respiratory health in COPD patients. Philip and Hafizurrachman [9] investigated the effect of pursed lip breathing on oxygen saturation levels in COPD patients. Their results corroborate our findings of improved respiratory outcomes, particularly in oxygenation, further validating the effectiveness of breathing techniques as a simple yet effective intervention. Sharaf *et al.* [10] also assessed the effects of pursed lip breathing on physiological parameters, finding significant improvements. This is consistent with our results, where we noted substantial reductions in dyspnoea and improved bio-physiological parameters, reinforcing the role of breathing exercises in managing COPD symptoms.

The pre-and post-test levels of bio-physiological parameters among patients with COPD in the interventional and control groups were compared. The interest is to compare the pre-test and post-test levels of bio-physiological parameters among patients with COPD in the interventional and control groups. In the present study, both groups exhibited similar distributions of dyspnea levels and respiratory issues before the intervention, with no significant statistical differences. However, following the intervention, the interventional group demonstrated significant improvements compared to the control group. Mean scores for respiratory issues in the interventional group notably improved, decreasing from 7.45 to 4.80, representing a significant mean difference of 2.65 (p = 0.001). In contrast, the control group showed minimal improvement, with mean scores slightly decreasing from 7.35 to 7.05. These findings suggest that the respiratory care modalities effectively enhanced respiratory health outcomes in the interventional group, reducing dyspnea severity and improving other biophysical parameters related to respiratory health. Thus, H1 was accepted, confirming a significant difference in post-test levels of biophysical parameters between the interventional and control groups. Another objective was to find the association between the post-test level of bio-physiological parameters among the patients with COPD and their selected demographic and clinical variables. This objective focused on finding the association between the post-test levels of biophysical parameters among COPD patients and their selected demographic and clinical variables. The study revealed significant correlations between mild dyspnea and several factors, including age, sex, regular treatment for comorbidities and family history of respiratory illness. Specifically, age and sex, along with family health history, were associated with normal to mild biophysical scores. Other variables did not significantly impact dyspnea or biophysical parameters, highlighting the importance of these demographic and health history factors in the prevalence and severity of mild dyspnea. The conversation focused on the need for individualized management of patients with COPD. Indeed, it recognized that factors such as age, gender, habits in treatment and family history related to general health characterize the severity of biophysical parameters. Recognition and intervention in these individual and familial health factors lead to better outcomes, further emphasizing the importance of customized treatments of respiratory care services for patients suffering from breathing diseases. For this reason, H2 was accepted and a significant association between the post-test levels of the selected physiological parameters in both interventional and control groups and their selected demographic and clinical variables was confirmed.

## Conclusion:

Tailored respiratory care modalities significantly enhanced physiological parameters in COPD patients. These findings strongly advocate integrating such modalities into standard COPD management, as they can dramatically improve overall health outcomes and quality of life.

## Figures and Tables

**Table 1:** Physiological parameters assessment scale

**S. No**	**Features observed**	**Score**		
		**Score - 0**	**Score - 1**	**Score - 2**
1)	Respiratory Rate	25 to 30/m	31 to 40/m	> 40/min
2)	Pulse rate	80 to 100/m	101 to120/m	> 120/min
3)	Nasal flaring	NIL	Unilateral	Bilateral
4)	Body temperature	37.c	37.c to 39.c	>39.c
5)	Chest retraction	None	Just Visible	Marked
6)	Use of accessory Muscles	None	Moderate	Maximum
7)	Cough	None	Non productive	Productive
8)	Breathing sound	Normal	Occasional rales	Crepitating
9)	O2 Saturation	98% to 100%	95% to 97 %	<95%

**Table 2:** Comparison of dyspnoea scores between interventional and control group

**Dyspnoea score**	**Group**				**Mean difference**	**Mann Whitney U-test**
	**Interventional**		**Control**			
	**Mean**	**SD**	**Mean**	**SD**		
Pre-test	2.6	1.5	2.53	1.6	0.07	Z=0.25 p=0.81(NS)
Post-test	1.62	1.1	2.42	1.6	0.8	Z=2.45 p=0.02*(S)
*p≤0.001 very high significant
S= significant. NS- Non-significant

**Table 3:** Comparison of bio-physiological parameters score between interventional group and control group

**io physiological parameters score**	**Group**				**Mean difference**	**Mann Whitney U-test**
	**Interventional**		**Control**			
	**Mean**	**SD**	**Mean**	**SD**		
Pre-test	7.45	3.21	7.35	4.62	0.1	Z=0.21 p=0.83(NS)
Post-test	4.8	4.71	7.05	4.23	2.25	Z=2.25 p=0.05*(S)
*p≤0.001 very high significant
S= significant. NS- Non-significant
